# Dysregulation of microRNA and Intracerebral Hemorrhage: Roles in Neuroinflammation

**DOI:** 10.3390/ijms22158115

**Published:** 2021-07-29

**Authors:** Hisham Kashif, Dilan Shah, Sangeetha Sukumari-Ramesh

**Affiliations:** Department of Pharmacology and Toxicology, Medical College of Georgia, Augusta University, Augusta, GA 30912, USA; hkashif@augusta.edu (H.K.); dilshah@augusta.edu (D.S.)

**Keywords:** ICH, microRNA, miR, neuroinflammation

## Abstract

Intracerebral hemorrhage (ICH) is a major public health problem and devastating subtype of stroke with high morbidity and mortality. Notably, there is no effective treatment for ICH. Neuroinflammation, a pathological hallmark of ICH, contributes to both brain injury and repair and hence, it is regarded as a potential target for therapeutic intervention. Recent studies document that microRNAs, small non-coding RNA molecules, can regulate inflammatory brain response after ICH and are viable molecular targets to alter brain function. Therefore, there is an escalating interest in studying the role of microRNAs in the pathophysiology of ICH. Herein, we provide, for the first time, an overview of the microRNAs that play roles in ICH-induced neuroinflammation and identify the critical knowledge gap in the field, as it would help design future studies.

## 1. Introduction

Intracerebral hemorrhage (ICH) is one of the most devastating subtypes of stroke, accounting for 10–15% of all stroke cases [1]. The mortality rate of ICH is 45.4% within one year of initial ictus [2] and around 74% of ICH survivors remain functionally dependent at one year after the onset of symptoms [3]. Moreover, the incidence of ICH is expected to increase due to population aging and the spreading use of anticoagulants [4]. Despite recent advances in preclinical research, effective treatments for ICH have not been found, which partly attributes to the lack of understanding of the complex pathophysiology of ICH. 

Intracerebral hemorrhage refers to the spontaneous extravasation of blood in the brain parenchyma and typically occurs in the basal ganglia, thalamus and cerebral lobes [5]. Patients with hypertension or cerebral amyloid angiopathy have a higher disposition to ICH, mainly because hypertension and cerebral amyloid angiopathy contribute to structural and functional vascular abnormalities and make blood vessels more vulnerable to rupture [6]. ICH often results in severe brain damage, which is categorized into primary and secondary brain injuries. The primary injury is mostly physical damage to the brain resulting from the rapid formation as well as the mass effect of the hematoma. The secondary injury evolves as an overlapping continuum to primary damage and results from extravasated blood components and associated neurotoxicity. The mechanisms of secondary brain damage include, but are not limited to, neuroinflammation, oxidative stress, apoptosis, and excitotoxicity [7,8]. Among these, a growing body of evidence reveals the potential of therapeutically targeting neuroinflammation to modulate both brain injury and repair after ICH. 

MicroRNAs (miRNA, miR) are small conserved non-coding single-stranded RNA that can be transcribed from intronic, intergenic, and protein-coding regions in the genome by RNA polymerase II [9] or RNA polymerase III [10]. The promoters for transcription are found either upstream or downstream of miR sequences [11] and the primary transcription product (pri-miRNA) can code for several miRs or only one miR. The pri-miRNA undergoes enzymatic processing into the pre-miRNA transcript, a 60 to 70 nucleotides long hairpin structure, which subsequently gets translocated from the nucleus [12,13,14]. The cytoplasmic RNase III Dicer cleaves the pre-miRNA into a 19 to 23 nucleotides long mature miRNA [15,16,17,18]. Functionally, microRNAs control gene expression by binding with the 3′ untranslated region (UTR) of the target messenger RNA (mRNA). As a consequence, the target mRNA will either be degraded or preserved and translated later [19,20]. A single miR could regulate hundreds or even thousands of transcripts and modulate multiple signaling cascades [21,22]. Notably, miRs contribute to various biological processes such as cell differentiation, proliferation, metabolism, death, and innate immune response [23]. Though the vast majority of miRs are intracellular, there are miRs in the extracellular body fluids that play a role in cell-cell signaling [24,25]. However, most of the circulating miRs are non-functional and passively released from cells through apoptosis or necrosis [26].

The mammalian brain expresses the highest number of microRNAs [27] in comparison to other organs. Recent evidence reveals several distinct mechanisms by which microRNAs regulate brain disease [28,29] and pathological conditions are often associated with dysregulated miR expression. Neuroinflammation is characterized by increased activation of microglia (the innate immune cells of the central nervous system (CNS)) and the subsequent release of pro-inflammatory and anti-inflammatory mediators. The inflammatory brain response also involves the interplay between the cells within the CNS and in the periphery [30]. miRs are ideal candidates to be explored for the treatment of various neuroinflammatory conditions since they can have a unique cellular or tissue expression profile in response to neuroinflammation [31]. Moreover, the aberrant miR expression could result in altered inflammatory responses in the microglia [32]. Consistently, Dicer (a protein that is critical for the biogenesis of miR)-deficient microglia exhibited enhanced activation in response to inflammatory stimuli [33]. Additionally, miRs can be released from cells such as neurons facilitating neuronal-glial communication and thereby contributing to neuroinflammation [34,35,36,37]. Taken together, miR is emerging as a promising therapeutic target to modulate neuroinflammation and improve neurological function. Herein, we provide, for the first time, an overview of microRNAs that play a role in neuroinflammatory response after ICH. 

## 2. MicroRNA and ICH-Induced Neuroinflammation

The applications of miR as potential therapeutic agents and diagnostic markers continue to evolve as more and more studies investigate the involvements of miR in various pathologies. The last four years have seen a surge in research questioning the role of various miRs in the pathophysiology of ICH. Functional studies were often conducted using miR mimics, which supplement miR expression, or with miR antagonists, which downregulate miR expression. ICH results in profound neuroinflammation and herein, we discuss the miRs that could serve as potential regulators of ICH-induced neuroinflammation, a complex pathological process contributing to neuroprotective and neurodegenerative effects. 

### 2.1. miR-144

Altered miR-144 expression is associated with a multitude of neuropathologies and is implicated in cellular proliferation [38], inflammatory response [39,40], and β-amyloid deposition [41]. The expression of miR-144 was studied in preclinical models of ICH. To this end, the experimental induction of ICH in mice by the brain injection of autologous blood was associated with significantly enhanced miR-144 levels in the perihematomal brain region at 24 h post-ICH [39]. Further, the brain administration of miR-144 inhibitor improved neurobehavioral outcomes with a concomitant reduction in cerebral edema and RNA levels of proinflammatory cytokines such as IL-6, IL-1β, and TNF-α, implicating a role of miR-144 in neuroinflammatory response and brain injury after ICH [39]. In addition, inhibition of miR-144-3p, a member of the miR-144 family, attenuated cerebral edema and neurobehavioral deficits in a blood model of ICH in rats via formyl peptide receptor 2 (FPR2), a key regulator PI3K/AKT signaling and inflammation [42]. 

Free hemoglobin is regarded as a potent inducer of ICH-induced oxidative [43] as well as inflammatory brain damage [44]. After intracerebral hemorrhage (ICH), the erythrocytes that accumulate in the brain parenchyma undergo lysis and subsequently hemoglobin gets released. Employing in vitro studies, Wang et al., 2017 [40] demonstrated that hemoglobin could enhance miR-144 expression in microglia, the inflammatory cells of the CNS, a possible mechanism by which miR-144 levels are increased after ICH. In addition, Wang et al., 2017 [40] showed that inhibition of miR-144 attenuates hemoglobin-mediated microglial inflammatory response and autophagy via the mTOR (mammalian Target of Rapamycin) signaling pathway, further implicating its role in inflammatory brain damage. However, a recent report employing the collagenase injection model of ICH did not demonstrate a significant increase in miR-144 expression in the perihematomal brain region, though there was an upward trend in comparison to control [45]. Additionally, the genetic deletion of miR-144 along with miR-451 augmented neuroinflammation and brain injury after ICH implicating a neuroprotective role of the miR-144/451 cluster [45]. Altogether, the data indicate the need to conduct further longitudinal studies establishing the efficacy of miR-144 as a viable therapeutic target.

### 2.2. miR-155

The expression of miR-155, a marker of inflammation [46,47,48] was studied in ICH patients and animal models of ICH. Of note, the serum level of miR-155 was significantly elevated in patients with ICH and was associated with hematoma volume, a critical determinant of neurological outcomes after ICH [49]. Moreover, serum level of miR-155 was positively correlated with 6 months-mortality and unfavorable outcomes after ICH implicating its potential to serve as a prognostic marker [49]. Though studies are yet to be conducted to determine whether there is an elevated serum level of miR-155 in animal subjects with ICH, the collagenase injection mouse model of ICH showed an increase in miR-155 in the brain tissue 3 days post-injury [50]. Furthermore, dexamethasone-mediated attenuation of the expression of proinflammatory cytokines such as IFN-β, IL-6, and TNF-α after ICH in mice was associated with a reduction in miR-155, implicating its role in neuroinflammation [50]. Moreover, a recent preclinical study reported the efficacy of miR-155 inhibitors in improving acute neurological outcomes after ICH [51]. In line with this observation, the genetic inhibition of miR-155 attenuated ischemic brain damage with a reduction in the release of proinflammatory cytokines in a mouse model of MCAO and attenuated glucose deprivation/oxygenation-induced proinflammatory cytokine expression in vitro [52]. Moreover, increased expression of miR-155 in glioma patients is associated with poor prognosis [53], and miR-155 is implicated in the inflammatory response associated with several neurodegenerative disorders [54,55]. Altogether, further studies are highly required to explore its potential as a therapeutic target after ICH. 

### 2.3. miR-222

miR-222 has been shown to be associated with cardiovascular diseases and various inflammatory conditions. A recent study by Bai and colleagues [56] found increased brain expression of miR-222 at 72 h post-injury in a blood injection model of ICH in mice. Furthermore, cerebroventricular administration of miR-222 inhibitor reduced the brain water content, release of proinflammatory mediators, and acute neurological deficits in mice after ICH implicating its critical role in secondary brain damage [56]. In line with this observation, miR-222 mimics significantly augmented ICH-induced acute neurological deficits in mice. Bai and colleagues [56] also demonstrated that erythrocyte lysate is a potent inducer of miR-222 expression in cultured microglia and inhibition of miR-222 significantly attenuated erythrocyte lysate-induced microglial release of proinflammatory cytokines, further implicating its role in neuroinflammation. Additionally, studies from the same group revealed that miR-222 regulates the release of inflammatory cytokines by negatively targeting integrin subunit β8 (ITGB8), a member of the integrin family, which mediates cell-extracellular matrix interactions. Of note, ITGB8 up-regulation has shown to attenuate inflammation in vitro [56], whereas its deletion enhances the formation of dysplastic blood vessels and hemorrhage [57,58]. However, studies need to be conducted to determine the precise molecular mechanisms of miR-222-mediated neurotoxic effects after ICH. 

### 2.4. miR-145

miR-145 expression was found to be significantly increased in the blood plasma of ICH patients in comparison to control [59]. Therefore, it may have the potential to be a biomarker of ICH warranting further investigation. Notably, apart from its well-documented role in tumor suppression [60], miR-145 is implicated in IL-4 and IFNβ mediated brain immune responses [31,61] and regulates the release of TNF-α from adipocytes [62]. In addition, miR-145 regulates SMAD-3, an activator of the anti-inflammatory mediator transforming growth factor-β (TGFβ). SMAD-3 is a transcription factor, which plays a role in neuronal apoptosis after ICH [63]. Therefore, studies need to be conducted determining the functional role of miR-145 in the blood plasma or in systemic inflammatory responses, which is often associated with brain injuries and its expression level changes in the brain after ICH. 

### 2.5. miR-494

Both preclinical and clinical studies suggest that microglia/macrophage-mediated inflammatory response plays an important role in hemorrhage-induced brain damage [64,65]. ICH results in very profound activation of microglia/macrophages, highly plastic cells, which display diverse phenotypes [66]. To this end, M1 microglia/macrophage release proinflammatory mediators and contribute to brain damage, whereas M2 microglia/macrophage generate anti-inflammatory cytokines and promote brain recovery. Hence, the molecular mechanisms that decrease M1 activation and augment M2 activation of microglia/macrophages are identified and characterized to improve neurological outcomes. Recent studies document the role of microRNA in the regulation of microglia/macrophage polarization [67]. To this end, treatment with miR-494 mimics elevated the M1 macrophage polarization, with an increase in brain water content and neurological damage at 3 days post-ICH in mice suggesting a detrimental role of miR-494 after ICH [67]. Additionally, when microglia were treated with miR-494 mimics, it augmented M1 polarization in vitro [67]. Moreover, miR-494 expression levels were increased in the perihematomal brain area at day 1, day 3, and day 5 post-ICH, a time course that exhibits a prominent inflammatory response after ICH in mice [67]. Mechanistically, miR-494 targets the E3 ubiquitin protein ligase, NRDP1, a protein that plays a critical role in macrophage polarization [67]. miR-494 mimics downregulated the expression of NRDP1 and that was associated with an increase in ICH-induced inflammation, neurological deficits, and cerebral edema [67]. In contrast, Changlong Zhou et al. documented that NRDP1 promotes inflammation after ICH [68]. These conflicting results demand further investigation validating the functional role of NRDP1 after ICH. 

### 2.6. miR-223

Reduced expression of miR-223, a hematopoietic specific microRNA [69] with crucial functions in myeloid lineage development [70], was observed in the ipsilateral brain region in mice after ICH [71]. Functionally, miR-223 exerts acute neuroprotection after ICH as the intracerebroventricular administration of miR-223 mimics attenuated neurological deficits and cerebral edema in mice at 48 h post-ICH [71] and that was associated with a reduction in the expression of NLRP3 and proinflammatory cytokines [71]. NLRP3 is a key component of NLRP3 inflammasome, a protein complex that plays a critical role in ICH-induced neuroinflammation [72]. In vitro studies further confirmed the anti-inflammatory role of miR-223. Along these lines, miR-223 mimics significantly attenuated erythrocyte lysate-induced inflammatory response in microglia with a reduction in NLRP3 expression [71]. Furthermore, NLRP3 mRNA contains conserved miR-223 binding sites in its 3′ UTR, implicating that miR-223 could be a direct regulator of NLRP3 expression [71], warranting further investigation. In contrast to preclinical brain samples of ICH, miR-223 expression was elevated in the plasma of patients in the acute phase of ICH in comparison to control, suggesting its potential to serve as a diagnostic marker [59]. Moreover, serum exosomal levels of miR-223 were associated with the occurrence, severity, and short-term outcomes of ischemic stroke [73]. Therefore, the functional role or consequence of elevated plasma expression of miR-223 in the ICH patient population and its implications in neurological outcomes need to be elucidated. 

### 2.7. miR-7

In normal physiology, miR-7 regulates the development of the pituitary gland, optic nerve system, and cerebral cortex [74]. In contrast, dysregulated miR-7 expression is associated with a variety of pathological conditions, such as cellular metastasis [75], α-synuclein accumulation [76], amyloid peptide accumulation [77], and apoptosis [78]. Moreover, miR-7 is a highly expressed miR in the mammalian brain [74]. Zhang and colleagues [79] demonstrated a significant decrease in miR-7 expression in the perihematomal brain region in patients diagnosed with ICH and in a preclinical rat model of ICH. Employing an in vitro approach, they also showed that the proinflammatory mediator TLR4 [80] is a direct target of miR-7 [79], implicating a role of miR-7 in inflammation. Additionally, mimics of miR-7 were effective in reducing the lipopolysaccharide-induced inflammatory response in microglia [79]. Consistently, another research group has documented that miR-7 agomirs attenuated neurological function score and brain water content in a preclinical model of ICH [81]. Despite its role in inflammation, miR-7 could also reduce 1-methyl-4-phenylpyridinium (MPP)-induced neuronal apoptosis through inhibition of NF-κB [82] and regulation of the mTOR signaling pathway [83]. Altogether, further studies are needed to determine the mechanism by which miR-7 downregulation occurs after ICH and its effect on ICH-induced oxidative neuronal damage and long-term neurological deficits. 

### 2.8. miR-let-7a

miR-let-7a is regarded as an immunomodulatory microRNA and mainly regulates anti-inflammatory signaling [84]. Consistently, Yang and colleagues [85] reported that miR-let-7a promotes M2 microglia polarization in a preclinical model of ICH. Along these lines, miR-let-7a expression was decreased in the perihematomal brain region at 3 days post-ICH and intracerebroventricular administration of miR-let-7a mimics attenuated pro-inflammatory cytokine expression and augmented anti-inflammatory cytokines with a significant improvement in acute neurological outcomes in mice. It was also demonstrated that the anti-inflammatory effects of miR-let-7a after ICH were mediated partly via CKIP-1 (Casein Kinase 2 Interacting Protein-1, also known as PLEKHO1), a protein that plays a role in cellular apoptosis [86], and microglia polarization [87]. However, further studies need to be conducted with CKIP-1 knockout animals to establish the role of CKIP-1 in miR-let-7a mediated neuroprotection after ICH. Furthermore, though decreased serum level expression of miR-let-7a is observed in ICH patients in comparison to controls [88], its functional significance is yet to be established. 

### 2.9. miR-21-5p

miR-21 is one of the highly expressed miR in many mammalian cell types [89] and its expression is often altered in pathological conditions [90,91,92,93]. Of note, miR-21 can regulate the Akt [94] and/or ERK/MAPK pathways [95] and the expression of inflammatory mediator, Toll-like 4 receptor (TLR4) [96]. Moreover, miR-21 has emerged as a key regulator of the anti-inflammatory signaling in macrophages [97]. Apart from its role in immune responses, miR-21 is a critical participant in necroptosis [98], a type of cell death observed in preclinical models of ICH [99]. In the blood injection model of ICH in rats, adenovirus-mediated overexpression of miR-21-5p attenuated proinflammatory response at 72 h post-ICH, with a reduction in brain water content and neurological deficits implicating that miR-21-5p exerts neuroprotection [100]. In contrast, in aged animals, genetic knockdown of miR-21-5p attenuated neuronal apoptosis, neuroinflammation, and neurobehavioral deficits at 24 post-ICH [101], suggesting that miR-21-5p is a contributor to ICH-induced brain damage. Importantly, serum miR-21-5p levels were elevated in elderly patients in the acute phase of ICH in comparison to healthy subjects [101]. Additionally, there was a positive correction between elevated serum miR-21-5p levels and National Institutes of Health Stroke Scale (NIHSS) scores and clinical outcomes after ICH [101]. Altogether, there exists a discrepancy in the functional roles of miR-21-5p after ICH and hence, further studies are required to validate the potential of miR-21-5p as a therapeutic target and determine whether aging modulates the functional roles of miR-21-5p after ICH.

### 2.10. miR-23a-3p

Nrf2 is an antioxidant transcription factor that plays roles in both oxidative as well as inflammatory brain damage [102]. miR-23a-3p regulates cell proliferation and metastasis through inhibition of PTEN [103], a regulator of Nrf2 [104]. Notably, in a rat blood-injection model of ICH, the expression of miR-23a-3p was increased in the perihematomal brain region 3 days after ICH [105], implicating its possible role in the pathophysiology of ICH. Consistently, the genetic inhibition of miR-23a-3p attenuated ICH-induced neurodegeneration, ferroptosis (an iron-dependent cell death), and the release of proinflammatory cytokines in rats [105]. Of note, miR-23a-3p antagomir-mediated neuroprotection after ICH was associated with an induction of HO-1, a critical downstream target of Nrf2 [106]. Given the potential of Nrf2 activators in improving neurological outcomes after ICH [102], further studies are required to characterize the precise molecular mechanisms of miR-23a-3p-induced brain injury after ICH. 

### 2.11. miR-23b

miR-23b, which plays roles in cell migration [107], proliferation [108], growth [109], and MAPK signaling pathway [110], is largely understudied in neuropathological conditions. In a rat model of ICH, the expression of miR-23b was downregulated in the perihematomal brain region at day 1, day 3, and day 5 post-ICH [111]. In addition, hemin, a hemoglobin metabolite and critical modulator of ICH-induced secondary brain damage [65], attenuated the expression of miR-23b in BV2 mouse microglial cells in vitro [111], suggesting a possible mechanism for its downregulation in preclinical models of ICH. Functionally, lentiviral-mediated genetic overexpression of miR-23b reduced inflammatory response both in vitro and in vivo [111]. Additionally, genetic overexpression of miR-23b significantly attenuated neurological deficits at day 1, day 3, and day 5 post-ICH [111]. Though in vitro studies report inositol polyphosphate multikinase (*IPMK*), which promotes Toll-like receptor-induced inflammation, as a target of miR-23b [111], future studies are warranted elucidating the molecular mechanisms of miR-23b-mediated neuroprotection in preclinical models of ICH. 

### 2.12. miR-124

miR-124 is one of the most abundantly expressed miR in the adult mammalian brain and accounts for 25–48% of all brain microRNAs [112]. Despite its role in neuronal differentiation [113], maturation [114], and survival during CNS development, the role of miR-124 in normal adult brain function is yet to be determined. However, in neuropathological conditions, miR-124 is implicated in cell survival [115], apoptosis [116], autophagy [117], neuroinflammation [118] and oxidative damage [119]. Of note, miR-124 is regarded as a potential biomarker of tissue injury [120] and cerebral infarction [121]. Consistently, in the collagenase injection model of ICH and human ICH patients, miR-124 is significantly upregulated in the plasma during the acute phase and downregulated in the recovery phase [122]. Given its prominent induction in human plasma in the acute phase of ICH, miR-124 may serve as a promising biomarker for the diagnosis of ICH [122]. Furthermore, the brain expression of miR-124 was significantly elevated in the rat model of ICH in the acute phase in comparison to sham [122], suggesting that brain damage may result in its release into the plasma. In contrast to the collagenase injection model, a separate study using the blood-injection model reported significantly reduced expression of miR-124 in the perihematomal region after ICH in mice [123]. Moreover, erythrocyte lysate reduced microglial expression of miR-124, suggesting the blood extravasation must be contributing to its altered brain expression after ICH [123]. Additionally, miR-124 mimics significantly attenuated M1 but increased M2 markers in mice, implicating the role of miR-124 in microglial polarization both in vitro and in vivo [123]. The anti-inflammatory effects of miR-124 mimics in mice were associated with improved neurological outcomes and reduced cerebral edema and apoptotic cell death. Additionally, the inhibitors of miR-124 had an opposite effect and its administration led to increased ICH-induced inflammatory damage in mice. Based on the target prediction program TargetScan analysis, a potential mediator that was identified as a target of miR-124 was CCAAT/enhancer-binding protein alpha (CEBP-α), a regulator of microglia polarization [123]. However, the study did not present data derived from one critical experimental group (ICH + vehicle) and hence, caution should be made in drawing conclusions from it. Moreover, given the discrepancy in the brain expression levels of miR-124 in preclinical models of ICH, further studies are highly warranted to validate its role in neuroinflammation. Additionally, a recent study in aged animal subjects reports that administration of miR-124 antagomir attenuated iron accumulation [124], implicating a neurotoxic potential of miR-124. Notably, increased serum miR-124 levels were correlated with poor neurologic scores in aged ICH patients [124]. Overall, despite the potential of miR-124 inhibitors or agonists in modulating neurological outcomes in preclinical models of ICH, further studies are required to establish its efficacy as a potential therapeutic target after ICH. 

### 2.13. miR-126-3p

Reduced expression of miR-126-3p, a regulator of inflammation [125], was observed in the serum, perihematomal area, and hematoma in a collagenase injection model in rats at 24 h post-ICH [126]. The preclinical studies reported a neuroprotective role of miR-126-3p after ICH because the administration of miR-126-3p mimics reduced cerebral edema, blood-brain-barrier permeability, microglia activation, neuronal apoptosis, and acute neurological deficits in rats through regulation of PIK3R2 (phosphoinositide-3-kinase regulatory subunit 2) and Akt [126]. Additionally, there is a negative correlation between serum miR-126 and perihematomal edema in ICH patients [88]. However, further studies need to be conducted elucidating the mechanisms of miR-126-3p-mediated neuroprotection after ICH, as inflammation plays an important role in cerebral edema development after ICH. 

### 2.14. miR-129-5p

In contrast to other miRs, the expression of miR-129-5p was studied at a non-acute time point post-ICH and there was a reduction in its expression at 7 days and 14 days post-ICH in the brain tissue of rats [127], possibly suggesting a role of miR-129-5p to modulate long-term neurological outcomes. Intravenous administration of liposomes expressing miR-129-5p attenuated ICH-induced induction of high mobility group box-1 (HMGB1), an endogenous ligand of TLR4 [128,129], implicating its pivotal role in neuroinflammation [127]. Consistently, miR-129-5p mimic prevented NF-kB signaling in autoimmune diseases by inhibiting TLR4 or TLR2-HMGB1 pathway [130,131] and attenuated neuroinflammation after ischemia-reperfusion by inhibiting HMGB1 and the TLR3-cytokine pathway [132]. However, neurological outcomes studies are yet to be conducted to demonstrate the efficacy of targeting miR-129-5p after ICH. 

### 2.15. miR-132

Owing to its dual roles in immune response and neuronal functions, miR-132 is named “NeurimmiR” [133]. Along these lines, miR-132 has a critical role in brain development, synapse formation, and synapse maturation [134,135]. Additionally, miR-132 promotes the cholinergic anti-inflammatory pathway by targeting acetylcholinesterase, culminating in the reduced release of proinflammatory cytokines [136]. The anti-inflammatory potential of miR-132 is explored widely across various pathological conditions. Consistently, a study by Zhang et al. [137] employing a mouse model of ICH reported that lentiviral-mediated overexpression for miR-132 in the mouse brain striatum attenuated neurological deficits, cerebral edema, ICH-induced changes in blood-brain barrier permeability, neuroinflammation, and neuronal apoptosis at day 3 post-ICH. It was also proposed that miR-132-mediated neuroprotection after ICH could be attributed to reduced brain levels of acetylcholinesterase [137]. However, the study has not evaluated whether ICH modulates miR-132 expression in the mouse brain and the long-term effects of miR-132 overexpression. Apart from acetylcholinesterase (AChE) [137], miR-132 also targets IRAK4 [138], a regulator of proinflammatory signaling [138]. Altogether, further studies are highly required before miR-132 can be considered a therapeutic target. 

### 2.16. miR-140-5p

Mounting evidence indicates that TLR4 is a promising therapeutic target for ICH. To this end, the increased expression of TLR4 was associated with poor outcomes in ICH patients [139]. Moreover, TLR4 inhibitors are effective in reducing secondary brain damage after ICH [140]. Of note, recent studies document TLR4 as a target of miR-140-5p, a tumor suppressor in various human cancers [141,142], and a reduction in the brain expression levels of miR-140-5p was observed after ICH in rats [143]. Therefore, miR-140-5p could serve as a potential candidate to be considered to improve outcomes after ICH. Along these lines, administration of miR-140-5p mimics, prior to ICH induction, attenuated neurological deficits and neuroinflammation in rats [143]. Given the critical role of TLR4 in ICH-induced neuroinflammation [144,145], studies are highly warranted to test whether post-injury administration of miR140-5p improves outcomes after ICH. 

### 2.17. miR-146a

miR-146a dysregulation is observed in relation to a large number of neuropathological conditions where it mainly modulates inflammatory response [146]. Based on preclinical studies, it is reported that miR-146a-5p exerts neuroprotection after ICH [100]. To this end, the adenovirus-mediated overexpression of miR-146a in the rat brain attenuated proinflammatory cytokine levels with an improvement in neurological outcomes at day 3 post-ICH [100]. Subsequently, a study by Qu and colleagues using a preclinical model reported the downregulation of miR-146a in the perihematomal area at 48 h post-ICH and validated the anti-inflammatory potential of miR-146a after ICH [147]. Mechanistically, miR-146a-mediated neuroprotection was associated with a reduction in the brain expression of TRAF6 [147], which is a key regulator of NF-kB and NLRP3 inflammasome signaling [148]. Additionally, overexpression of miR-146a attenuated the apoptosis of hippocampal neurons after ICH in rats [149], further implicating its potential to be a viable therapeutic target after ICH, warranting future studies. 

### 2.18. miR-152

miR-152, a tumor suppressor microRNA that was mostly associated with cell survival [150,151,152], was downregulated in the perihematomal brain tissue of rats on day 1, day 3, and day 5 post-ICH [153]. In vitro studies revealed that hemin, a hemoglobin metabolite that accumulates in the brain after ICH, is a regulator of miR-152 expression [153]. Moreover, genetic overexpression of miR-152 attenuated brain water content, neuronal death, hematoma size, and neurological deficits in rats, implicating a neuroprotective role of miR-152 [153]. Mechanistically, miR-152 regulated NLRP3 inflammasome activation by modulating the expression of thioredoxin interacting protein (TXNIP) after ICH [153]. Additionally, lentiviral-mediated overexpression of miR-152 attenuated the serum levels of proinflammatory cytokines [153], suggesting its possible and unexplored role in systemic inflammation. Additionally, miR-152 was significantly downregulated in the serum of ICH patients compared with controls [154]. Overall, dysregulation of miR-152 contributes to the pathophysiology of ICH and could be considered as a novel therapeutic target, warranting further investigation. 

### 2.19. miR-181c

miR-181c, an independent prognostic indicator for glioblastoma multiforme [155], had altered expression levels in the serum samples of patients diagnosed with various brain pathologies [156,157,158] and was associated with irregular cell proliferation, migration [155,159], Th17 cell differentiation [160], and amyloid-beta plaque buildup [161]. Employing in vitro studies, it was demonstrated that thrombin, a proteolytic enzyme that contributes to ICH pathology, significantly downregulated the expression of miR-181c in human microglia [162]. Additionally, miR-181c could modulate thrombin-mediated NF-κB target gene expression in vitro by negatively regulating NF-κB activity [162], suggesting that miR-181c is a potential candidate to be considered to modulate thrombin-induced microglial activation after ICH. A subsequent study by Lu and colleagues [163] reported a significant decrease in expression levels of miR-181c in the plasma samples of ICH patients and ipsilateral brain samples derived from rats at an undisclosed time-point after ICH. Furthermore, miR-181c mimics improved neurological function in rats, whereas miR-181c inhibitor exacerbated neurological deficits after ICH by modulating neuronal apoptosis. However, future studies are highly needed to define the unexplored role of miR-181c in ICH-induced neuroinflammation. 

### 2.20. miR-183-5p

miR-183-5p is a negative regulator of heme oxygenase-1 [164] an enzyme that plays critical roles in iron homeostasis and oxidative and inflammatory brain damage after ICH [165]. Though HO-1 augments acute brain damage after ICH in mice [165], the precise functional role of HO-1 in microglia/macrophages, the prominent cell type that expresses HO-1 after ICH, is yet to be determined. In the collagenase induction model of ICH in mice, reduced brain expression of miR-183-5p was observed, and administration of its mimics attenuated ferrous deposition, ROS (reactive oxygen species) production in the brain, microglial/macrophage activation, proinflammatory cytokine levels, and neurobehavioral defects after ICH implying that miR-183-5p exerts acute neuroprotection [164]. Moreover, miR-183-5p agonists attenuated oxidative neuronal toxicity in vitro [166]. Overall, future studies are warranted to characterize further the molecular mechanisms of miR-183-5p-mediated neuroprotection and the functional role of miR-183-5p in long-term neurological outcomes after ICH.

### 2.21. miR-194-5p

Reduced expression of miR-194-5p, a miR related to inflammatory responses [167,168] is observed in the perihematomal brain tissue in rats at 6, 12, 24, and 48 h post-ICH [169]. Functionally, miR-194-5p agomir attenuated neurological deficits and brain water content via modulating the expression of tumor necrosis factor receptor-associated factor 6 (TRAF6), a protein directly involved in NLRP3 inflammasome expression and activation after ICH [169]. Given the role of NLRP3 inflammasome in ICH-induced brain damage [72], additional studies are required to validate the therapeutic potential of miR-194-5p. 

## 3. Therapeutic and Diagnostic Implications of microRNA

**Figure 1:**
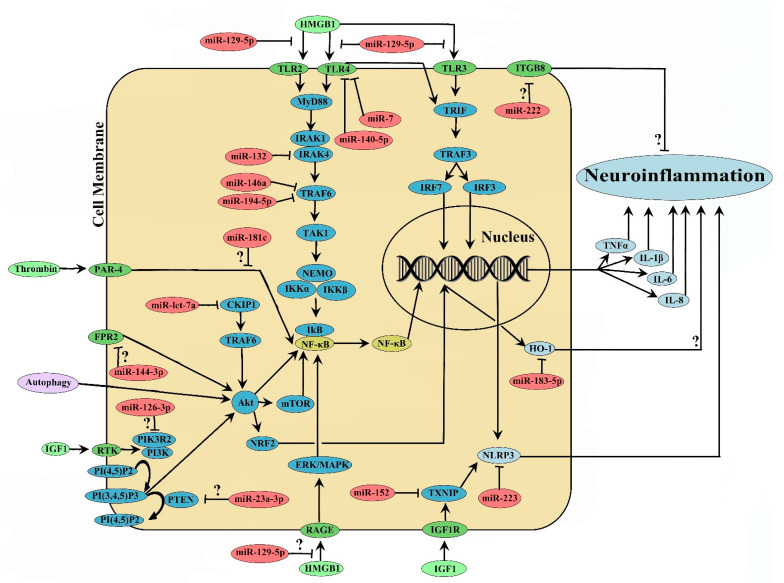
Mechanism(s) by which miRs possibly regulate neuroinflammation after ICH.

Regulation of gene transcription is the most fundamental component of cellular responsiveness to environmental changes. To this end, one of the evolutionarily conserved proteins that connects external stimuli with the gene expression changes is NF-κB [170], a master regulator of inflammation [171,172,173] and there are a variety of exogenous NF-κB inhibitors in clinical trials or the market [174]. Notably, NF-κB plays a critical role in brain damage after ICH [175] and is regarded as a potential target of therapeutic intervention. Moreover, NF-κB activation in the perihematomal brain region is an independent predictor of the patient outcome at 6 months after ICH [176]. As illustrated in Figure 1, miRs that play roles in the neuroinflammatory response after ICH could be associated with regulation of NF-κB activation. 

NF-κB can be activated through MyD88/IRAK1/IRAK4/TRAF6 [177], TRIF [178], PI3K/Akt [179], and MAPK/ERK pathways [180]. Upon activation, NF-κB translocates to the nucleus, binds to DNA, and promotes the transcription of proinflammatory cytokines. The pathways most regulated by miR in the context of ICH-induced neuroinflammation are TLR (Toll-like receptor)/MyD88/IRAK1/IRAK4/TRAF6 signaling and Akt signaling. Along these lines, miR-140-5p, miR-7, miR-146a, miR-194-5p, miR-132, and miR-129-5p, could modulate neuroinflammation possibly via regulating NF-κB activation (Figure 1). Among these, miR-140-5p [143], miR-7 [79], and miR-129-5p [127] attenuated ICH-induced neuroinflammation possibly through the inhibition of TLR4 signaling while miR-146a [147], and miR-194-5p [169] exerted anti-inflammatory effects potentially through TRAF6 inhibition and miR-132 exerted similar effects possibly through IRAK4 inhibition [138]. In addition, NLRP3, a downstream target of NF-κB [181], can also be regulated by miR. To this end, miR-223 [71] and miR-152 [153] could negatively regulate NLRP3 inflammasome and improve acute neurological outcomes after ICH. Furthermore, miR-144-3p [42] and miR-126-3p [126] potentially regulate neuroinflammation through modulation of Akt signaling, a known upstream activator of NF-kB [179]. In addition, miR-let-7a [85] also could regulate Akt signaling and hence neuroinflammation via CKIP-1, a potential upstream regulator of Akt [182] (Figure 1). However, it would be worth investigating whether miR-mediated effects occur independently of NF-κB activation. Though the functional roles of M1 or M2 microglia/macrophages after ICH are yet to be defined, miR-494 augmented M1 microglia activation and neurological deficits [67], whereas miR-let-7a-mediated neuroprotection after ICH was associated with an increase in M2 microglia/macrophages polarization [85]. NRDP1 [67] or CKIP-1 [85] was partly responsible for M1 and M2 polarization, respectively. However, the functional role of NRDP1 on ICH-induced inflammation is largely controversial [67,68], warranting further investigation. Taken together, miR dysfunction after ICH contributes to microglial polarization and cytokine release, critical brain responses that play roles in secondary brain damage and brain recovery. Besides neuroinflammation, miR-23a-3p [105] and miR-183-5p [164] may also regulate oxidative brain damage, further implicating the potential of targeting miR in improving neurological outcomes after ICH. 

Additionally, the members in the same family of miR may exert different functional roles. To this end, miR-23a-3p has a neurodegenerative role [105], while miR-23b has a neuroprotective role after ICH [111]. Although they are both derived from the same double-stranded precursor, they may have different functional targets. 

The preclinical studies that were conducted to elucidate the functional roles of miR employed mostly young animal subjects. Of note, age is an independent predictor of ICH outcomes [183]. miR-21-5p and miR-124 had neuroprotective roles in young animals [100,123], but exerted neurodegenerative effects in old animal subjects after ICH [101,124]. Therefore, additional studies need to be performed to better elucidate the relationship between advanced age and the functional roles of miR. Additionally, the preclinical studies should incorporate female subjects to determine the influence of sex in miR-regulated brain damage after ICH.

The currently established methods of miR detection and quantification include reverse transcription quantitative polymerase chain reaction (RT-qPCR; TaqMan), SplintR-qPCR, and miREIA. Although RT-qPCR is a widely used method, it comes with the error-prone step of converting miRNA to cDNA and difficulty in generating a reliable calibration curve [184]. In contrast, SplintR-qPCR uses a hybridization and ligation step prior to RT-PCR, whereas miREIA employs unique hybridization and specific antibody to DNA/RNA hybrids [184,185] and both approaches generate more precise results. However, despite the challenges in the detection and quantification of miRs, serum/plasma miRs may serve as promising diagnostic markers. Along these lines, apart from their possible role in inflammation, miR-145 [59], miR-223 [59], miR-155 [49], and miR-152 [154] may serve as biomarkers of ICH, but it is not clear whether they may help discriminate hemorrhagic stroke from ischemic stroke, requiring additional studies. Additionally, further investigation needs to be conducted elucidating the source of circulating miRNA whether they are derived from the injured brain or arise systemically after ICH. 

As RNA-based therapeutics enter clinical practice [186,187], it is further affirmed that miR can serve as a potential target for therapeutic intervention. Clinical therapeutic strategies that are being considered to manipulate miR expression include the use of oligonucleotides (miR mimics or artificial antagonists). Given the poor pharmacokinetic properties and insufficient efficiency of oligonucleotides in clinical trials [188], chemical modifications such as phosphorothioates, 2′-methoxyethyl-nucleotides, and locked nucleic acids (LNA) with an efficient delivery system or packaging are critical for clinical applicability. To this end, extracellular vesicles (EVs), a heterogeneous group of small membrane vesicles, including exosomes, can serve as an efficient vehicle for synthetic oligonucleotide or miR delivery [189], facilitating cell-cell communication because of the natural availability, stability, biocompatibility, and low immunogenicity of EVs [190]. Furthermore, stem cell-derived exosomes can be engineered to carry exogenous miRs [191] targeting specific cells [188,192].

Given the potential of miRs in modulating various pathological processes, several phase 1 clinical trials are either completed (pending results) or underway testing the safety and tolerability of miR-based treatment strategies. For instance, oligonucleotides targeting miR-155 and miR-21 have completed Phase 1 testing for certain lymphomas [NCT02580552; clinicaltrials.gov], and Alport syndrome [NCT03373786; clinicaltrials.gov], respectively. Though the ongoing clinical trials on various neuropathological conditions are largely focused on testing whether miR can serve as a biomarker, a clinical trial is currently recruiting patients to evaluate the efficacy of administration of exosomes overexpressing miR-124 in improving outcomes after acute ischemic stroke (NCT03384433; clinicaltrials.gov). However, despite the progress in preclinical studies, clinical trials are yet to be conducted testing the therapeutic potential of miRs in improving outcomes after ICH. 

## 4. Conclusions

Altogether, miR dysfunction contributes to the pathophysiology of ICH. To this end, miR-223, miR-7, miR-let-7a, miR-23b, miR-126-3p, miR-132, miR-140-5p, miR-146a, miR-152, miR-181c, miR-183-5p and miR-194-5p promote neuroprotective effects, whereas miR-222, miR-494, miR-23a-3p confer neurodegenerative effects in preclinical models of ICH. Given the potential of miR as viable therapeutic targets, further studies are required to elucidate the molecular mechanisms of miR dysregulation after ICH. Additionally, studies need to be conducted in a sex- and age-independent manner to fully extrapolate the efficacy of targeting miR to improve neurological outcomes after ICH.

## Data Availability

Not applicable.

## Abbreviations

AChE	acetylcholinesterase
Akt	protein kinase B
BBB	blood brain barrier
CEBP-α	CCAAT/enhancer-binding protein alpha
CKIP-1	Casein Kinase 2 Interacting Protein-1, also known as PLEKHO1
CNS	central nervous system
ERK	extracellular signal-regulated kinase
FPR2	formyl peptide receptor 2
Hb	hemoglobin
HMGB1	high mobility group box-1
HO-1	heme oxygenase 1
ICH	intracerebral hemorrhage
IFNβ	interferon beta
IGF1	Insulin Like Growth Factor 1
IGF1R	Insulin Like Growth Factor 1 Receptor
IkB	inhibitory Kappa B
IKK	inhibitor of nuclear factor-κB (IκB) kinase
IL-1β	interleukin 1 beta
IL-4	interleukin 4
IL-6	interleukin 6
IPMK	inositol polyphosphate multikinase
IRAK1	interleukin 1 Receptor Associated Kinase 1
IRAK4	interleukin 1 Receptor Associated Kinase 4
IRF3	Interferon Regulatory Factor 3
IRF7	Interferon Regulatory Factor 7
ITGB8	integrin subunit β8
LNA	locked nucleic acid
MAPK	mitogen activated protein kinase
MCAO	middle cerebral artery occlusion
miR	microRNA(s)
MPP	1-methyl-4-phenylpyridinium
mTOR	mammalian target of Rapamycin
MyD88	Myeloid differentiation primary response 88
NEMO	NF-kappa-B essential modulator
NF-κB	nuclear factor kappa light chain enhancer of activated B cells
NFE2L2	nuclear factor, erythroid 2 like 2
NLR	nod like receptor
NLRP3	NLR family pyrin domain containing 3
NRDP1	neuregulin receptor degradation protein-1
Nrf2	nuclear factor erythroid 2-related factor 2
PI(3,4,5)P3	Phosphatidylinositol (3,4,5)-trisphosphate
PI(4,5)P2	Phosphatidylinositol 4,5-bisphosphate
PI3K	phosphoinositide 3 kinase
PIK3R2	phosphoinositide 3 kinase regulatory subunit 2
pri-miRNA	primary long miRNA transcript
PTEN	phosphatase and tensin homolog
RAGE	Receptor for advanced glycation endproducts
RNA	ribonucleic acid
RNA Pol II	RNA polymerase II
RNA Pol III	RNA polymerase III
ROS	reactive oxygen species
RT-qPCR	reverse transcription quantitative polymerase chain reaction
RTK	receptor tyrosine kinase
Th17	T helper 17 cells
TLR	toll like receptor
TLR2	toll like receptor 2
TLR3	toll like receptor 3
TLR4	toll like receptor 4
TNF-α	tumor necrosis factor alpha
TRAF3	TNF receptor-associated factor 3
TRAF6	tumor necrosis factor receptor-associated factor 6
TRIF	(Toll/interleukin-1 receptor) domain-containing adaptor inducing IFNβ
TXNIP	thioredoxin interacting protein
UTR	untranslated region
